# Supplementary material for: A cluster of acidic residues in the cytoplasmic domain of SARS-CoV-2 Spike is required for virion-incorporation and infectivity
Source: PLoS One. 2026 Mar 12;21(3):e0340644. doi: 10.1371/journal.pone.0340644 (PMC12981473; doi:10.1371/journal.pone.0340644)

S1 Raw Images. Unprocessed western blot and SDS-PAGE gel images

Unprocessed western blot images corresponding to Figure 3 (experiment 1).

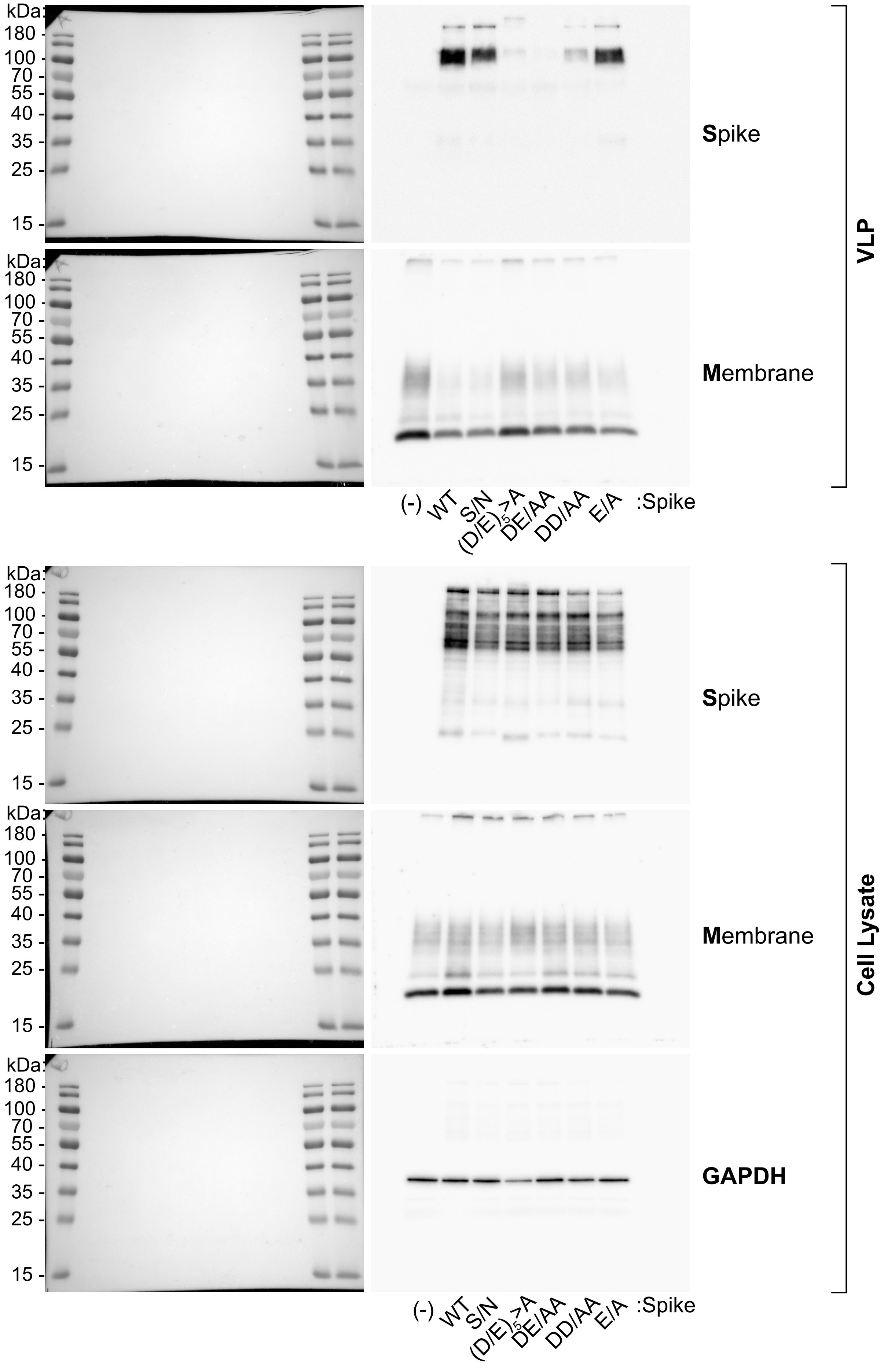

S1 Raw Images. Unprocessed western blot and SDS-PAGE gel images

Unprocessed western blot images corresponding to Figure 3 (experiment 2).

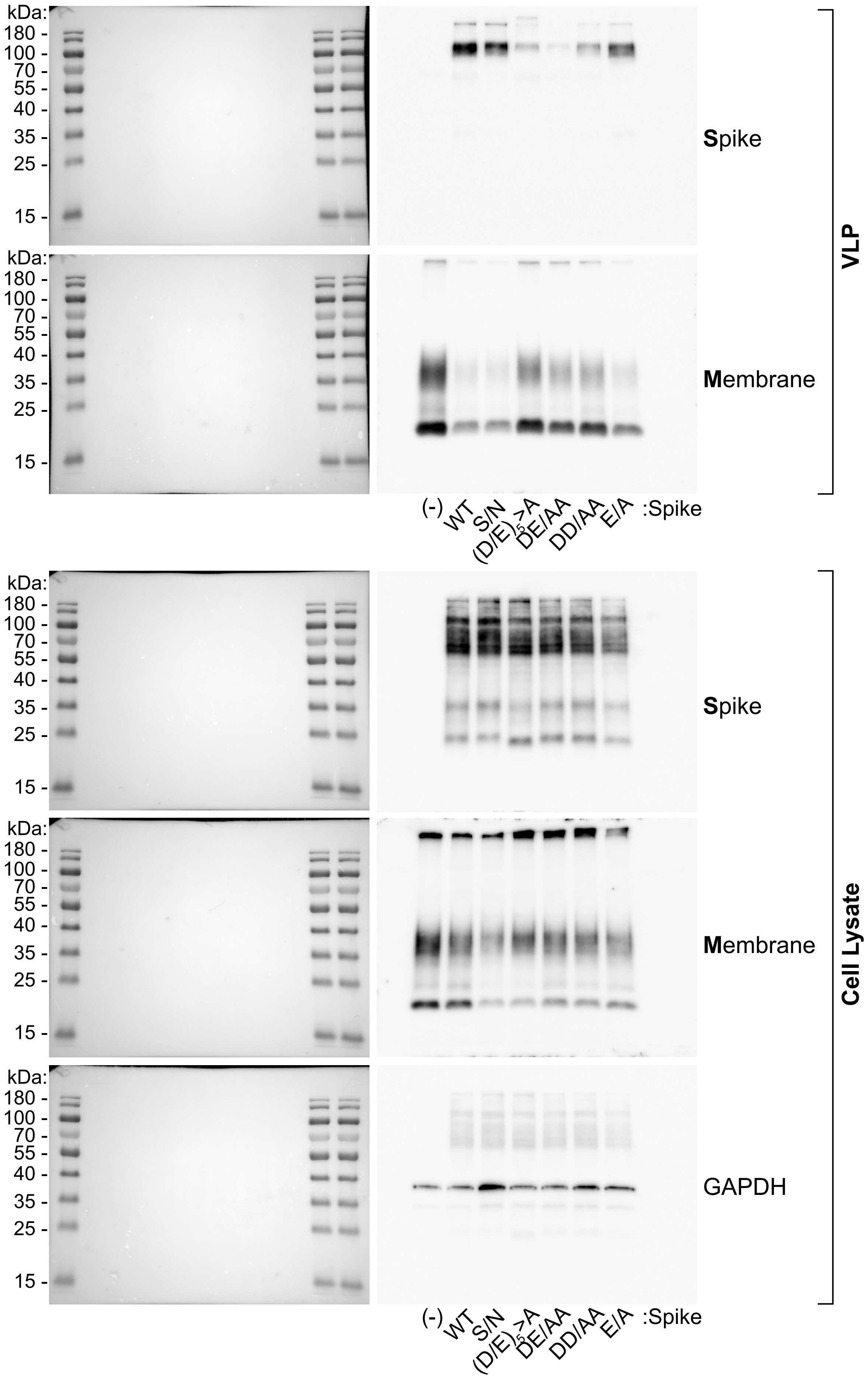

S1 Raw Images. Unprocessed western blot and SDS-PAGE gel images

Unprocessed images of coomassie gel staining from GST-pulldown experiments (corresponding to Figure 5)

A

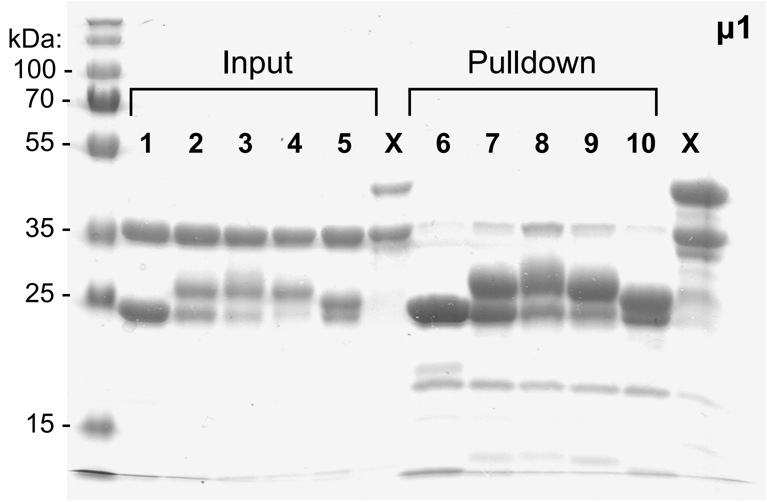

μ1/2 + GST-Spike CD:

Input:
1. No Spike
2. Spike WT
3. Spike WT + CK-II
4. Spike S/N + CK-II
5. Spike (D/E_5)>A + CK-II

Pulldown:
6. No Spike
7. Spike WT
8. Spike WT + CK-II
9. Spike S/N + CK-II
10. Spike (D/E_5)>A + CK-II

B1

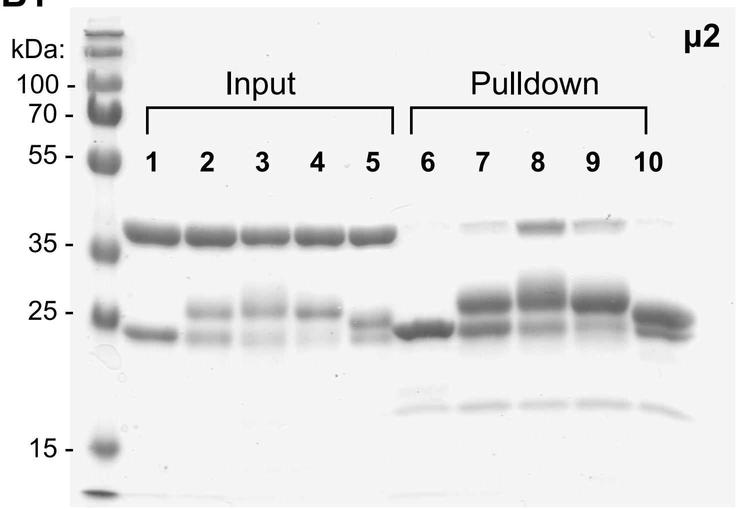

B2

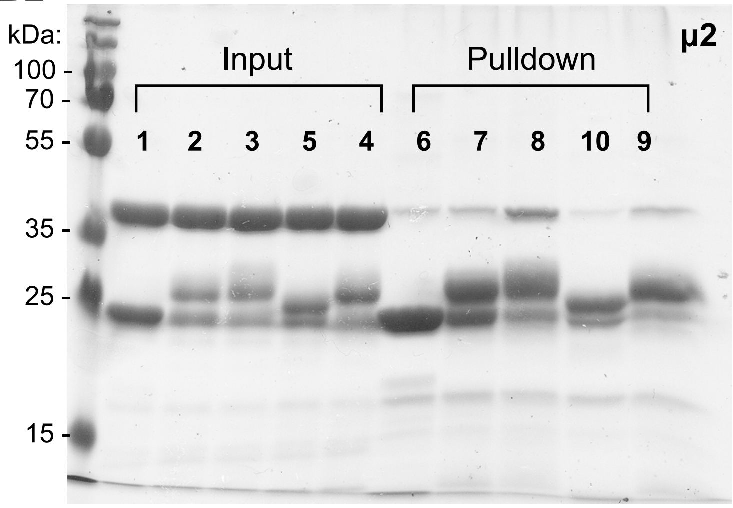

Supplement: S1 Raw Images — Uncropped and unmerged colorimetric and chemiluminescence images from two separate western blot experiments are shown (corresponding to Fig 3). Chemiluminescent bands corresponding to Spike (100 kDa band) and Membrane (20 kDa band) were quantified from the two experiments. Also included are unprocessed images of coomassie blue-stained SDS-PAGE gels from GST-pulldown experiments of Spike CD with medium subunits of clathrin adaptors 1 and 2 (corresponding to Fig 5). Protein bands corresponding to Spike (100 kDa band) and Membrane (20 kDa band) were quantified from the two experiments. (PDF) [file pone.0340644.s001.pdf]
